# Synthesis and antiviral activities of spacer-linked 1-thioglucuronide analogues of glycyrrhizin

**DOI:** 10.3762/bjoc.8.79

**Published:** 2012-05-08

**Authors:** Christian Stanetty, Andrea Wolkerstorfer, Hassan Amer, Andreas Hofinger, Ulrich Jordis, Dirk Claßen-Houben, Paul Kosma

**Affiliations:** 1 Department of Chemistry, University of Natural Resources and Life Sciences-Vienna, Muthgasse 18, A-1190 Vienna, Austria; 2 Savira Pharmaceuticals GmbH, Veterinärplatz 1, A-1210 Vienna, Austria; 3 Institute of Applied Synthetic Chemistry, Vienna University of Technology, Getreidemarkt 9, A-1060 Vienna, Austria

**Keywords:** antiviral activity, carbenoxolone, glycyrrhizin, influenza A virus, thioglycoside, triterpene

## Abstract

The influenza virus infection remains a significant threat to public health and the increase of antiviral resistance to available drugs generates an urgent need for new antiviral compounds. Starting from the natural, antivirally active compound glycyrrhizin, spacer-bridged derivatives were generated with improved antiviral activity against the influenza A virus infection. Simplified analogues of the triterpene saponin glycyrrhizin containing 1-thio-β-D-glucuronic acid residues have been prepared in good yields by alkylation of 3-amino and 3-thio derivatives of glycyrrhetinic acid with a 2-iodoethyl 1-thio-β-D-glucopyranosiduronate derivative. The spacer-connected 3-amino derivatives were further transformed into N-acetylated and N-succinylated derivatives. The deprotected compounds containing these carboxylic acid appendices mimic the glycon part of glycyrrhizin as well as the hemisuccinate derivative of glycyrrhetinic acid, carbenoxolone. Antiviral activities of the compounds were determined in a biological test based on influenza A virus-infected cells, wherein the 3-(2-thioethyl)-*N*-acetylamino- and 3-(2-thioethyl)-thio-linked glucuronide derivatives were effective inhibitors with IC_50_ values as low as 54 µM.

## Introduction

**Figure 1:**
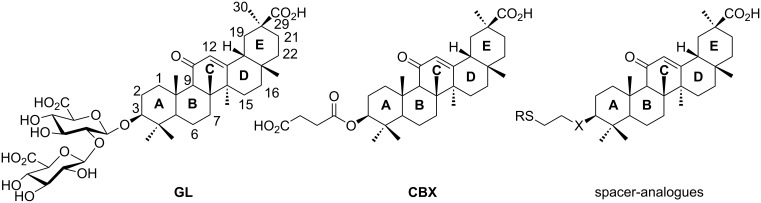
Structure of glycyrrhizin (**GL**), carbenoxolone (**CBX**), and spacer analogues.

The triterpene saponin glycyrrhizin (**GL**) and its aglycon glycyrrhetinic acid (**GA**) are the main triterpene components of licorice roots and harbor various pharmacological activities, including antitumor, anti-inflammatory, antioxidant and antiviral properties [1–2]. The antiviral activities have been reported to be directed against a broad spectrum of viruses comprising herpes-, corona-, alpha-, and flaviviruses, HIV, Epstein–Barr virus, influenza A virus (IAV), vaccinia and polio type I viruses as examples [3–8]. In particular anti-influenza virus activities have been described, although the underlying mechanisms of action are diverse [9–12]. In order to evaluate the potential antiviral properties of glycyrrhizin derivatives, we set out to modify the chemical environment in the vicinity of ring A of glycyrrhetinic acid. The envisaged modifications should assess the impact of acidic groups, which reside at the glucuronic acid residues in the β-(1→2)-linked disaccharide unit of **GL**, with respect to antiviral properties (Figure 1).

Previously, ring A modified derivatives of glycyrrhetinic acid were prepared containing 3-amino, 3-thio and 1-thio groups, allowing for an extension of the oleanolic acid unit with spacer groups as well as facile covalent attachment of carboxyalkyl groups by alkylation or acylation reactions [13–14]. For the compounds included in this investigation, the size of the spacer group was selected to closely match the formal distances between the A-ring and the carboxylic acid function present in the glucopyranosiduronic residues of the parent compound glycyrrhizin (**GL**) and in the hemisuccinate moiety of the glycyrrhetinic acid derivative carbenoxolone (**CBX**) [15] (Figure 1). For the sake of clarity, the nomenclature and numbering for the triterpene system as used throughout this report is illustrated in Figure 1. In order to enhance resistance against enzymatic cleavage by glucuronidases, the glucuronic acid residue was introduced through a stable thioglycosidic linkage.

## Results and Discussion

### Synthesis of 3-amino derivatives

**Scheme 1:**
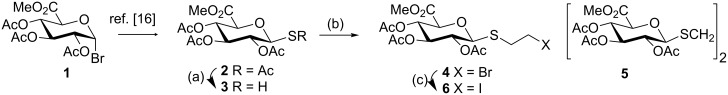
Synthesis of methyl 2-haloethyl 1-thio-glucuronide derivatives: (a) 1 M NaOMe, MeOH, −60 °C to −45 °C, 89% ; (b) Br(CH_2_)_2_Br, NaH, DMF, −5 °C (80% for **4**, 3% for **5**); (c) NaI, acetone, 4 °C, 16 h, 95%.

Commercially available methyl (2,3,4-tri-*O*-acetyl-D-glucopyranosyl)uronate bromide (**1**) was first reacted with potassium thioacetate in DMF to furnish the known 1-thioacetyl derivative **2** in 82% yield [16]. Subsequent treatment of **2** with sodium methoxide, under controlled conditions at low temperature (−60 °C → −45 °C), provided the corresponding glucuronyl 1-thiol **3** in 89% yield [17–18]. Reaction of **3** with an excess of 1,2-dibromoethane (3–4 equiv) in DMF in the presence of sodium hydride, with the strict exclusion of oxygen, afforded the 2-bromoethyl 1-thioglycoside **4** in 80% yield. Under these conditions formation of the bis-substitution product **5** (3%) and the disulfide oxidation product of 1-thiol **3** was observed in only very minor quanities. The corresponding 2-iodoethyl derivative **6** was prepared by a Finkelstein reaction from the bromoethyl 1-thioglycoside **4** in 95% yield (Scheme 1).

**Scheme 2:**
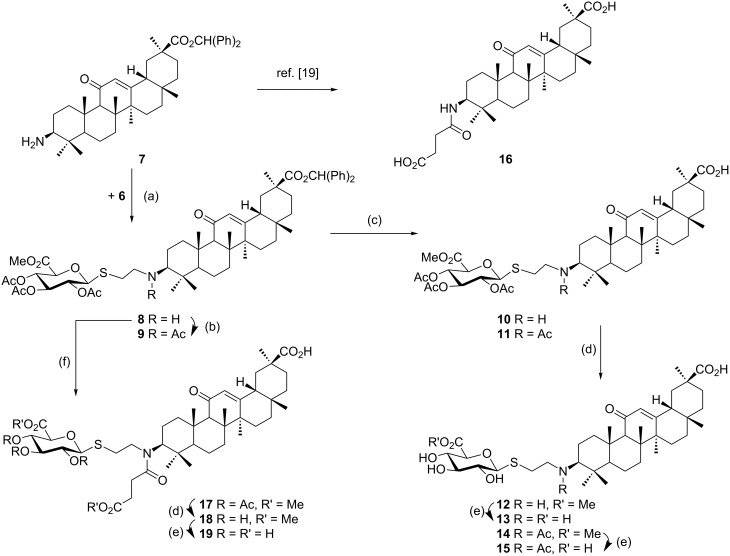
Synthesis of thioalkylglucuronide **GA** derivatives: (a) DMF, DIPEA, 45–50 °C, 16 h, 79%; (b) TEA, Ac_2_O, DCM, 0 °C, 2 h, 96%; (c) anisole, TFA, DCM, rt, 3 h (75% for **10**, 89% for **11**); (d) 0.1 M NaOMe, MeOH, 0 °C (89% for **12**, 97% for **14**, 99% for **18**); (e) 0.2 M NaOH, MeOH, rt, 3 h (91% for **13**, ~quant. for **15**, 99% for **19**); (f) MeO_2_C(CH_2_)_2_COCl in DCM, DIPEA, 0 °C to rt, 90 min, then anisole, TFA, DCM, 0 °C, 3 h, 83% for two steps.

The previously reported 3β-amino derivative of the diphenylmethyl ester of glycyrrhetinic acid **7** [19] was then used for the introduction of the spacer-extended 1-thio-glucopyranosiduronate residue. Coupling of the 3β-amino derivative **7** with two equivalents of the 2-iodoethyl glycoside **6** in DMF in the presence of Hünig base proceeded smoothly to provide the alkylated amine **8** in 79% yield without the formation of a bis-alkylated product (Scheme 2), whereas the use of the corresponding bromide **4** resulted in a significantly slower reaction. At elevated temperature, elimination leading to the corresponding hexenuronic acid derivative was observed (structure not shown). The secondary amino group in compound **8** was subjected to further derivatization by N-acetylation (triethylamine, acetic anhydride), which gave compound **9** in 96% yield. Deprotection of the ethyl 1-thio-glucuronide derivatives **8** and **9** was achieved by acid-catalyzed cleavage of the diphenylmethyl ester group with TFA/anisole (as carbocation scavenger), giving the monoacid derivatives **10** and **11** in 75% and 89% yield, respectively. Subsequent transesterification of **10** and **11** with methanolic NaOMe afforded the deacetylated methyl ester glucuronide derivatives **12** and **14**, respectively, which were finally fully deprotected by hydrolysis of the methyl ester group with 0.2 M methanolic NaOH to furnish the diacid derivatives **13** and **15**, in high overall yield.

**Figure 2:**
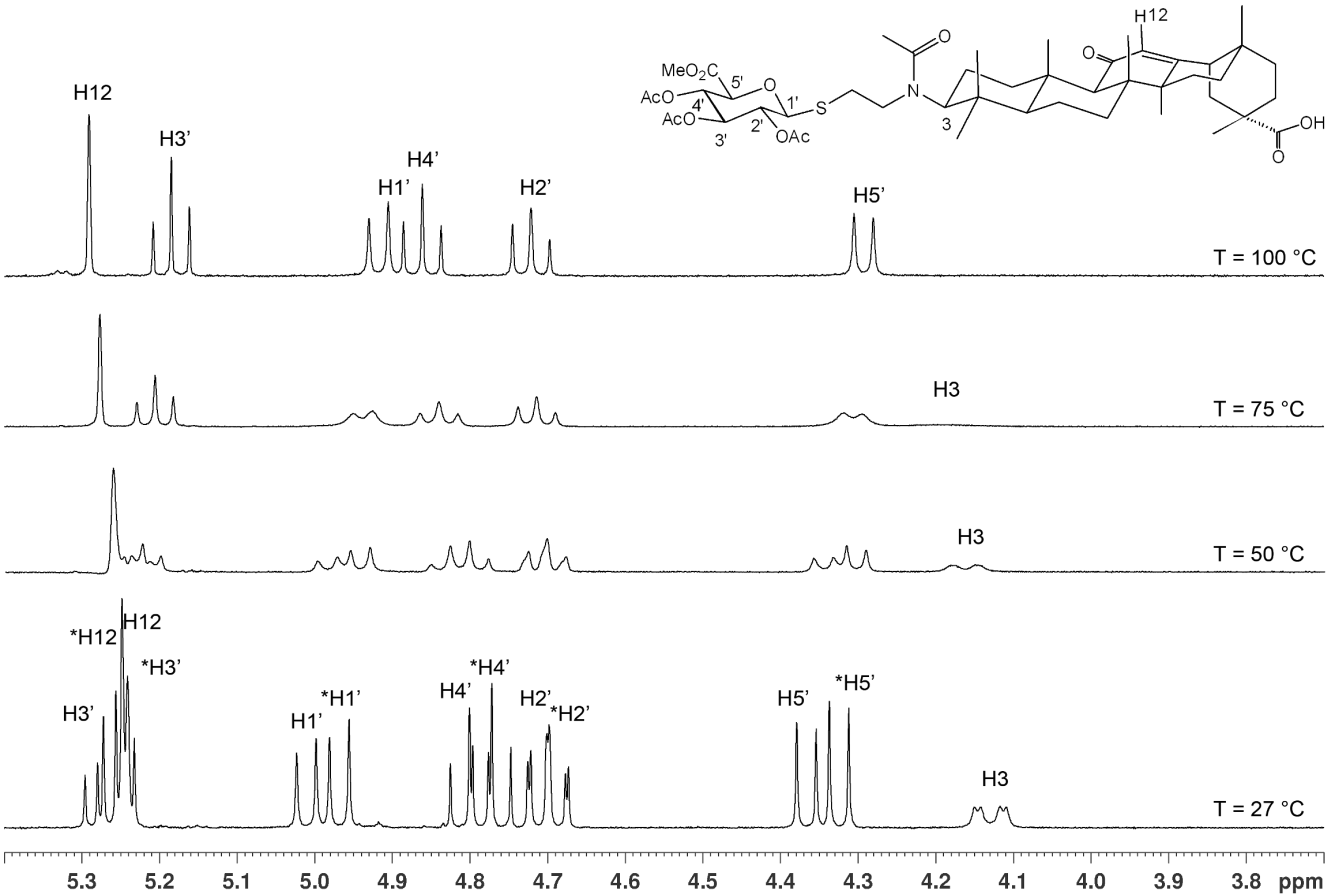
400 MHz ^1^H NMR expansion plots of the carbohydrate region of compound **11**, recorded at various temperatures.

A second series of compounds was designed as glucuronide-extended amide derivatives of the previously reported carbenoxolone analogue **16** [20–21]. Reaction of the 3-amino group of **8** with the monomethyl ester of succinic acid chloride in dichloromethane in the presence of Hünig base was followed by deblocking of the diphenylmethyl ester group to afford **17** in a combined yield of 83%. The acetyl groups of **17** were removed under Zemplén conditions to afford the dimethyl ester **18**. Alkaline hydrolysis of the ester groups eventually gave the triacid derivative **19** in good yield. In contrast to the straightforward chemical transformations, analysis of the NMR spectra of this series of compounds was challenging and the N-acylated derivatives **9**, **11**, **14**, **15** and **17**–**19** revealed complex spectra. Whereas the secondary amides of 3β-amino-glycyrrhetinic acid derivatives displayed coherent NMR signals [22–23], the tertiary amides frequently exist as mixtures of *cis*/*trans* rotamers leading to the duplication of signals [24–25]. The steric congestion at position 3 exerted by the two adjacent methyl groups may additionally contribute to the restricted rotation of the amide linkage. Thus, in the ^1^H NMR spectra of the *N*-acetyl- and *N*-succinyl derivatives, recorded at room temperature, two sets of signals in an approximate 6:4 ratio were observed. Reversible coalescence of these signal groups was observed upon heating a solution of the *N*-acetyl derivative **11** up to 100 °C in DMSO-*d*_6_ and subsequent cooling to room temperature (Figure 2), supporting the assignment of two signal data sets for two rotamers. The orientation of the amide carbonyl group in the rotamers also had a pronounced effect on the NMR chemical shifts of carbon and proton signals in ring A, which led to a shift of H-3 to lower field (δ 4.47) and to a shielding of C-3 (δ 59.9) for the minor isomer, whereas the inverse effects were seen for the major rotamer (δ for H-3 at 3.36 and δ for C-3 at 66.7). Similar, but less-pronounced effects were observed for the respective ^1^H and ^13^C signals at position 5 of ring A (see Supporting Information File 1).

### Synthesis of 3-thio derivatives

**Scheme 3:**
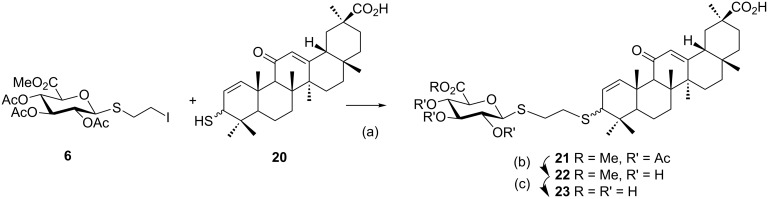
Synthesis of 3-thioether-bridged glucuronide derivatives: (a) K_2_CO_3_, acetone, 60%; (b) 0.8 M NaOMe, MeOH, 71%; (c) 0.2 M NaOH, MeOH, 95% .

Complementing the series of the 3β-amino series, related 3-thio derivatives were employed for the spacer elongation [14]. The previously reported 1,2-dehydro-3-thiol derivative **20** was subjected to alkylation with the 2-iodoethyl 1-thioglucuronide compound **6** in the presence of K_2_CO_3_ to furnish the thioether-bridged glucuronide triterpene **21** in 60% yield. Deprotection of **21** was performed in two steps as described for **10**, which furnished the methyl ester derivative **22** and the glucuronic acid compound **23**, respectively, in good yields (Scheme 3).

### Antiviral activities

For biological testing the compounds were dissolved in DMSO and diluted in cell culture medium to a maximum final DMSO concentration of 1%. For determination of the cytotoxicity of the compounds, MDCK cells were treated with compound concentrations ranging from 3.1 µM to 250 µM for 48 h and then cell viability was determined as a surrogate endpoint for cytotoxicity. Except for the aglycons of **GL**, namely glycyrrhetinic acid (**GA**) and the hemisuccinate carbenoxolone (**CBX**), which showed CC_50_ values (half-maximal cytotoxic concentration) of 7.4 µM and 17.8 µM, respectively, none of the compounds tested were toxic at concentrations up to 250 µM. **GL** was not toxic at 2500 µM (Table 1). The antiviral activity of the compounds was determined by infecting MDCK cells with influenza A H3N2 virus and treating them with different concentrations of compounds at the same time. Most compounds prevented virus-induced cytopathicity and restored cell viability compared to virus-infected control cells. The antiviral activity of glycyrrhizin (**GL**) against influenza and other viruses reported in the literature was confirmed in this study resulting in an IC_50_ of 1026 µM (Table 1). **GA** and **CBX** did not show any antiviral activity at nontoxic concentrations. Compounds **12**, **13**, **14**, **15**, **18** and **19**, which possess a (1-thio-β-D-glucopyranosyluronic acid)ethylamino substituent at position 3 of **GA** did not show toxicity at concentrations up to 250 µM, but showed enhanced antiviral activity compared to **GL**. Good activities were found for compounds **12** and **18** with IC_50_ values of 220.4 μM and 125 µM, respectively, being between 4.7-fold to 8-fold more active compared to the lead compound **GL**. The methyl ester **14** containing the N-acetylated spacer group was approximately 14-fold more active compared to **GL**, reflected by an IC_50_ value of 72.1 µM. The free acid **15** displayed an IC_50_ value in the same range as the methyl ester **14**. Replacement of the *N*-acetyl group of **14** with a methylsuccinyl group (**18**) did not improve the activity; however, the compound was still active with an IC_50_ value of 125 µM. The cleavage of both methyl ester groups of this compound resulted in compound **19** and led to the loss of antiviral activity up to the highest concentration tested (250 µM). Compound **16** containing only the *N*-succinyl substituent is a close analogue to **CBX** bearing an amide instead of the ester group. Unlike **CBX**, which is toxic at low concentrations, **16** was not toxic but inactive, indicating that the glucuronic acid moiety is associated with the antiviral properties. This was further supported by the data obtained with compound **23** containing a 1-thioethyl-linked glucuronic acid residue at position 3 of 1,2-dehydro-glycyrrhetinic acid. Compound **23** showed good activity with an even lower IC_50_ value of 54 µM compared to **15**, and exhibited also no cytotoxicity at the concentrations tested.

**Table 1:** Cytotoxic concentration 50% (CC_50_) of compound-treated uninfected cells and antiviral activities, shown as half-maximal inhibitory concentration (IC_50_) values of glycyrrhizin (**GL**) analogues.

Compound	Substructure	CC_50_ [µM]	IC_50_ [µM]

**GL**	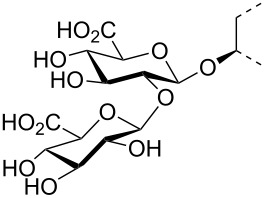	>2500	1026 ± 181
**GA**	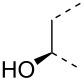	7.4 ± 0.05	nd^a^
**CBX**	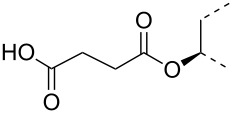	17.8 ± 2.3	nd^a^
**12**	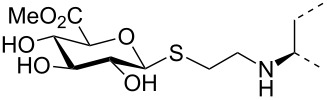	>250	220.4 ± 41
**13**	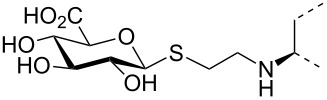	>250	nd (>250)^b^
**14**	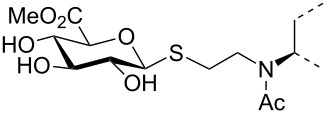	>75	72.1 ± 19.1^c^
**15**	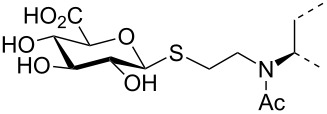	>250	87 ± 7.9
**16**	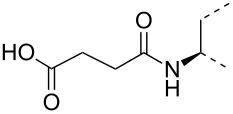	>250	nd (>250)^b^
**18**	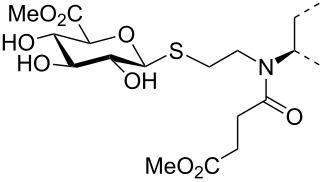	>250	125 ± 15.9
**19**	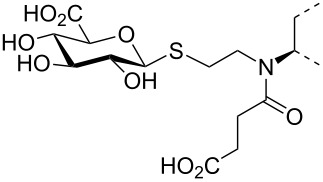	>250	nd (>250)^b^
**23**	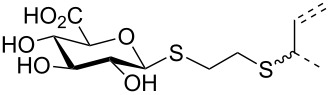	>250	54 ± 13

^a^nd not determined due to cytotoxicity; ^b^nd (>250) no detectable antiviral activity up to 250 µM; ^c^approximate value, since the compound precipitates at concentrations >75 μM.

## Conclusion

3-Amino and 3-thio derivatives of glycyrrhetinic acid served as versatile scaffolds for the attachment of glycosyl extensions through high-yielding alkylation reactions. The glucuronide derivatives linked via a thioethyl spacer group to the oleanolic acid unit exhibited no toxicity at concentrations up to 250 μM and significantly enhanced the anti-influenza virus activity of the natural triterpene glycoside glycyrrhizin.

## Supporting Information

File 1 Experimental details for the preparation of compounds **2**–**19** and **21**–**23** as well as the biological assays.
