# Supplementary material for: Synthesis and antiviral activities of spacer-linked 1-thioglucuronide analogues of glycyrrhizin
Source: Beilstein J Org Chem. 2012 May 8;8:705–11. doi: 10.3762/bjoc.8.79 (PMC3388857; doi:10.3762/bjoc.8.79)
Supplement: File 1 — Experimental details for the preparation of compounds 2–19 and 21–23 as well as the biological assays. [file Beilstein_J_Org_Chem-08-705-s001.pdf]

Supporting Information

for

Synthesis and antiviral activities of spacer-linked 1-thioglucuronide analogues of glycyrrhizin

Christian Stanetty[1§], Andrea Wolkerstorfer[2§], Hassan Amer[1], Andreas Hofinger[1], Ulrich Jordis[3], Dirk Claßen-Houben[2] and Paul Kosma*[1]

Address: [1]Department of Chemistry, University of Natural Resources and Life Sciences-Vienna, Muthgasse 18, A-1190 Vienna, Austria, [2]Savira Pharmaceuticals GmbH, Veterinärplatz 1, A-1210 Vienna, Austria and [3]Institute of Applied Synthetic Chemistry, Vienna University of Technology, Getreidemarkt 9, A-1060 Vienna, Austria

[§]These authors contributed equally to this work

Email: Paul Kosma - paul.kosma@boku.ac.at

* Corresponding author

Experimental details for the preparation of compounds 2–19 and 21–23 as well as the biological assays.

General methods

Compounds **7** and **20** were prepared as previously reported [1,2]. Compounds **12–15**, **18** and **19** were described elsewhere [3]. Whenever appropriate, solvents were purified and dried by standard procedures. Melting points were measured on a Büchi B-545 melting-point apparatus or a Kofler-type Reichert Thermovar hot-stage microscope and are uncorrected. Regarding NMR assignment and nomenclature, the carboxylic acid of **GA** was assigned as C29 and the adjacent methyl group as C30 (see Figure 1)

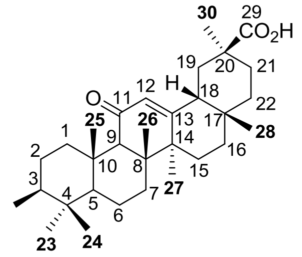

Figure 1: Numbering scheme of the oleanane aglycon.

Signals of rotamers are designated with * symbols. NMR spectra were recorded at 297 K in the solvent indicated, with a Bruker AC 300 and a Bruker DPX 400 spectrometer employing standard Bruker NMR software. Spectra were referenced to tetramethylsilane by calibration with the residual solvent peaks. Reactions were monitored by TLC on silica gel 60 F_{254} plates; spots were detected by UV light examination or visualized by spraying with anisaldehyde–sulfuric acid and heating. Normal-phase column chromatography was performed on silica gel 60 (230–400 mesh, Merck). HPLC–HRMS analysis was carried out from CH_3CN solutions (concentration: 1–10 mg/L) by using an HTC PAL system autosampler (CTC Analytics AG, Zwingen, Switzerland), an Agilent 1100/1200 HPLC with binary pumps, degasser and column thermostat (Agilent Technologies, Waldbronn, Germany) and Agilent 6210 ESI–TOF mass spectrometer (Agilent Technologies, Palo Alto, United States). Elemental analyses were provided by Dr. Theiner, Microanalytical Laboratory, University of Vienna.

Methyl (2,3,4-tri–*O*-acetyl-1-*S*-acetyl-1-thio-β-D-glucopyranosyl)uronate (**2**). To a solution of commercially available methyl (2,3,4-tri-*O*-acetyl-D-glucopyranosyl)uronate bromide (**1**) (10.0 g, 25.18 mmol, 1.00 equiv) in dry DMF (100 mL) potassium thioacetate (5.32 g, 46.58 mmol, 1.85 equiv) was added in several portions within 10 min at –10 °C. The reaction mixture was stirred at temperatures below 0 °C for 1 h and for 1 h at ~10 °C. Upon complete conversion (HPTLC, Hex/Et_2O 1:1) the reaction mixture was diluted with EtOAc (400 mL) and treated with satd. $NaHCO_3$ (200 mL) in a separatory funnel. The phases were separated and the aqueous layer was extracted with EtOAc. The combined organic layers were washed with water excessively and twice with brine, and then dried (Na_2SO_4) and evaporated. The crude material was crystallized from dry EtOH/EtOAc (60 mL + 5 mL) to give pure **2** (8.15 g, 82.5%) as colorless crystals. R_f 0.61 (SiO_2, Hex/EtOAc 1:1); mp 163–167 °C (EtOH/EtOAc); (lit. [4] mp 162 °C (dry EtOH)); ^{1}H NMR ($CDCl_3$) δ 2.03 (2×s, 6H, 2×OAc), 2.04 (s, 3H, OAc), 2.39, (s, 3H, SAc), 3.74 (s, 3H, OCH_3), 4.17 (d, $J_{4,5}$ = 9.9 Hz, 1H, H5), 5.11–5.23 (m, 2H, H2, H4), 5.29–5.37 (m, 2H, H1, H3).

Methyl 2,3,4-tri-*O*-acetyl-1-thio-β-D-glucopyranuronate (3). A solution of 1 M NaOMe in dry MeOH (3.1 mL, 3.1 mmol, 1.0 equiv) was added to a solution of thioacetate **2** (1.22 g, 3.10 mmol, 1.0 equiv) in dry MeOH/$CHCl_3$ 1:2 (15 mL) at a cooling-bath temperature of −60 to −55 °C (acetone/CO_2). As soon as precipitation of a white solid was observed, the reaction mixture was allowed to warm to −45 °C and was analyzed by TLC (Hex/EtOAc 1:1), indicating complete conversion. The reaction mixture was diluted with DCM (25 mL) and quenched with satd. NH_4Cl and water. The phases were separated, the aqueous layer was extracted with DCM (3x), the combined organic layers were washed with brine, dried (Na_2SO_4) and concentrated. The crude material was purified by column chromatography (SiO_2: 25 g, Hex/EtOAc 1:1) to afford pure target compound **3** (970 mg, 89%) as a white solid [4]. Storage under Ar at −25 °C is

recommended. R_f tailing to 0.3 (SiO_2, Hex/EtOAc 1:1); ^1H NMR ($CDCl_3$) δ 2.03 (2×s, 6H, 2×OAc), 2.06 (s, 3H, OAc), 2.40 (bs, 1H, SH), 3.76 (s, 3H, OCH_3), 4.02–4.09 (m, 1H, H5), 4.58 (d, $J_{1,2}$ = 10.0 Hz, 1H, H1), 4.97–5.03 (m, 1H, H2), 5.19–5.29 (m, 2H, H3, H4).

Methyl (2-bromoethyl 2,3,4-tri-*O*-acetyl-1-thio-β-D-glucopyranosid)uronate (4). A solution of thiol **3** (500 mg, 1.43 mmol, 1.00 equiv) and dibromoethane (1.07 g, 5.71 mmol, 4.00 equiv) in dry DMF (17 mL) was purged with Ar for 10 min before NaH (40 mg, 1.64 mmol, 1.15 equiv, washed with hexane) was added portionwise at −5 °C. Progress of the reaction was monitored by HPTLC (Hex/EtOAc 2:1). After the complete addition of NaH all starting material was consumed and the reaction mixture was quenched with half-satd. NH_4Cl (100 mL) at 0 °C and was extracted with DCM. The combined organic layers were washed with water (4×20 mL) and brine, dried (Na_2SO_4), evaporated and coevaporated from toluene twice. The crude material was purified by column chromatography (SiO_2: 20 g, Hex/EtOAc 3:1 → 1:2) to give pure target compound **4** (525 mg, 80.4%) as a white solid and small amounts of a more polar byproduct **5** (33 mg, 3.2%) as a second fraction. Analytical data of **4**: R_f 0.36 (Hex/EtOAc 2:1); mp 154–155.5 °C (needles, dry EtOH, softening from 150 °C); $[\alpha]_D^{20}$ −45.4 (*c* 0.4, $CHCl_3$); ^1H NMR ($CDCl_3$) δ 2.03 (s, 6H, 2×OAc), 2.07 (s, 3H, OAc), 2.95–3.05 (m, 1H, SCH_2), 3.16–3.26 (m, 1H, SCH_2), 3.52–3.60 (m, 2H, CH_2Br), 3.76 (s, 3H, OCH_3), 4.05 (d, $J_{4,5}$ = 9.4 Hz, 1H, H5), 4.59 (d, $J_{1,2}$ = 10.0 Hz, 1H, H1), 5.05 (*app.* t, *J* = 9.4 Hz, 1H, H2), 5.21 (*app.* t, *J* = 9.4 Hz, 1H, H4), 5.28 (*app.* t, *J* = 9.1 Hz, 1H, H3); ^{13}C NMR ($CDCl_3$) δ 20.5, 20.6, 20.7 (3×q, 3×COCH_3), 30.7 (t, CH_2Br), 32.2 (t, SCH_2), 53.0 (q, OCH_3), 69.1 (d, C4), 69.3 (d, C2), 72.7 (d, C3), 76.2 (d, C5), 83.7 (d, C1) 166.7 (s, C6), 169.29, 169.34, 167.0 (3×s, 3×*C*OCH_3); Anal. calcd: C, 39.40; H, 4.63; S, 7.01; found: C, 39.24; H, 4.67; S, 6.88.

Analytical data of **5**: R_f 0.14 (Hex/EtOAc 1:1); mp 171–174 °C (EtOH/EtOAc); $[\alpha]_D^{20}$ −52.3 (*c* 0.9, $CHCl_3$); ^1H NMR ($CDCl_3$) δ 2.03 (s, 12H, 4×OAc), 2.08 (s, 6H, 2×OAc), 3.77 (s, 6H, OCH_3), 2.81–2.93 (m, 2H, 2×SCH_2), 2.97–3.10 (m, 2H, 2×SCH_2), 4.11 (d, $J_{4,5}$ = 9.7 Hz, 2H, H5), 4.61 (d, $J_{1,2}$ = 10.0 Hz, 2H, H1,), 5.06 (*app*. t, *J* = 9.5 Hz, 2H, H2), 5.23 (*app*. t, *J* = 9.6 Hz, 2H, H4), 5.29 (*app*. t, *J* = 9.2 Hz, 2H, H3); ^{13}C NMR ($CDCl_3$) δ 20.5, 20.6, 20.7 (3×q, 3×$COCH_3$), 30.4 (t, S*C*H_2CH_2S, SCH_2*C*H_2S), 52.9 (q, OCH_3), 69.3 (d, C4), 69.4 (d, C2), 73.0 (d, C3), 76.1 (d, C5), 83.6 (d, C1) 166.9 (s, C6), 169.3, 169.4, 170.0 (3×s, 3×*C*OCH_3); Anal. calcd: C, 46.28; H, 5.27; S, 8.82; found: C, 46.03; H, 5.30; S 8.66.

Methyl (2-iodoethyl 2,3,4-tri-*O*-acetyl-1-thio-β-D-glucopyranosid)uronate (6). Bromide **4** (3.38 g, 7.39 mmol, 1.00 equiv) was dissolved in a 15% NaI solution (5.54 g, 36.9 mmol, 5.0 equiv) in acetone. The solution was purged with Ar for 15 min and stirred in an Ar atmosphere for 16 h at 4 °C in the dark, monitored by ^1H NMR. The reaction mixture was diluted with toluene (200 mL) and washed with water, brine, dried ($MgSO_4$) and concentrated. The crude material was applied onto SiO_2 (12 g) and purified by column chromatography (Hex/EtOAc 2:1 → 1:1) to give pure **6** (3.53 g, 94.7%, with traces of **4**) as a white solid. R_f 0.36 (Hex/EtOAc 2:1); mp 164–166 °C (Hex/EtOAc); $[\alpha]_D^{20}$ −45.7 (*c* 0.9, $CHCl_3$); ^1H NMR ($CDCl_3$): δ 2.03 (s, 6H, 2×OAc), 2.06 (s, 3H, OAc), 2.99–3.09 (m, 1H, SCH_2), 3.18–3.31 (m, 1H, SCH_2), 3.32–3.41 (m, 2H, ICH_2), 3.76 (s, 3H, OCH_3), 4.05 (d, $J_{4,5}$ = 9.4 Hz, 1H, H5), 4.58 (d, $J_{1,2}$ = 10.0 Hz, 1H, H1), 5.04 (*app*. t, *J* = 9.4 Hz, 1H, H2), 5.21 (*app*. t, *J* = 9.4 Hz, 1H, H4), 5.28 (*app*. t, *J* = 9.1 Hz, 1H, H3); ^{13}C NMR ($CDCl_3$) δ 2.9 (t, ICH_2), 20.4, 20.5, 20.6 (3×q, 3×CO*C*H_3), 33.1 (t, CH_2), 52.9 (q, OCH_3), 69.2 (d, C4), 69.4 (d, C2), 72.8 (d, C3), 76.2 (d, C5), 83.7 (d, C1) 166.7 (s, C6), 169.2, 169.3, 169.9 (3×s, 3×*C*OCH_3); Anal. calcd: C, 35.73; H, 4.20; S, 6.36; found: C, 35.64; H, 4.13; S, 6.23.

(3β,18β,20β)-3-{2-[Methyl (2,3,4-tri-*O*-acetyl-1-thio-β-D-glucopyranosyl)uronate] ethylamino}-11-oxo-olean-12-en-29-oic acid diphenylmethyl ester (8). A solution of iodide **6** (3.50 g, 6.94 mmol, 2.00 equiv) in DMF (11 mL) was purged with Ar for 15 min at rt. Amine **7** (2.20 g, 3.47 mmol, 1.0 equiv) and DIPEA (2.25 g, 17.35 mmol, 5.0 equiv) were added and the reaction mixture was stirred overnight at 45–50 °C external oil-bath temperature. HPLC analysis after 16 h indicated ~97% conversion of the starting material and the reaction mixture was allowed to reach rt. The solution was diluted with EtOAc, washed two times with satd. aq $NaHCO_3$, several times with water and once with brine. The organic layer was dried ($MgSO_4$) and coevaporated from toluene. The crude material was purified by MPLC (SiO_2: 220 g, DCM/MeOH 80:1 → 20:1) to give pure **8** (2.79 g, 79.5%) as white solid foam. R_f 0.30 ($CHCl_3$/MeOH 9:1 + 0.1% AcOH); $[\alpha]_D^{20}$ +75.0 (*c* 0.9, $CHCl_3$); ^1H NMR ($CDCl_3$) δ 0.67 (s, 3H, H28), 0.70 (m, 1H, H5), 0.75 (s, 3H, H24), 0.87–1.00 (m, 1H, H1b, H16b), 1.00 (s, 3H, H23), 1.09 (s, 3H, H26), 1.13 (s, 3H, H25), 1.10–1.20 (m, 1H, H15b), 1.18 (s, 3H, H30), 1.20–1.50 (m, 6H, H2b, H6b, H7b, H21b, H22a, H22b), 1.36 (s, 3H, H27), 1.55–1.75 (m, 4H, H2a, H6a, H7a, H19b), 1.79 (td, *J* = 13.6 Hz, *J* = 4.4 Hz, 1H, H15a), 1.99 (m, 5H, H3, H16a, H18, H19a, H21a), 2.02 (s, 6H, 2×OAc), 2.06 (s, 3H, OAc), 2.33 (s, 1H, H9), 2.66–2.82 (m, 3H, H1a, SCH_2, NCH_2), 2.85–2.95 (m, 1H, SCH_2), 3.00–3.10 (m, 1H, NCH_2), 3.75 (s, 3H, OCH_3), 4.03 (d, $J_{4,5}$ = 9.6 Hz, 1H, H5′), 4.60 (d, $J_{1,2}$ = 10.0 Hz, 1H, H1′), 5.06 (dd, $J_{1,2}$ = 10.0 Hz, $J_{2,3}$ = 9.1 Hz, 1H, H2′), 5.20–5.29 (m, 2H, H3′, H4′), 5.51 (s, 1H, H12), 6.94 (s, 1H, $OCHPh_2$), 7.25–7.41 (m, 10H, 10×PhCH); ^{13}C NMR ($CDCl_3$): δ 16.3 (q, C25), 16.5 (q, C24), 17.9 (t, C6), 18.7 (q, C26), 20.5 (q, $COCH_3$), 20.6 (q, $COCH_3$), 20.7 (q, $COCH_3$), 23.3 (q, C27), 24.4 (t, C2), 26.39, 2.41 (2×t, C15, C16), 28.25 (q, C30), 28.29 (q, C23), 28.37 (q, C28), 31.2, 31.3 (2×t, SCH_2, C21), 31.7 (s, C17), 32.8 (t, C7), 37.2 (s, C10), 37.5 (t, C22), 38.5 (s, C4), 39.9 (t, C1), 41.1 (t, C19), 43.1 (s, C20), 44.0 (s, C8), 45.3 (s, C14), 47.8 (t, NCH_2), 48.0 (d, C18), 52.9 (q, OMe), 56.1 (d, C5), 61.9 (d, C9), 65.9

(d, C3), 69.4 (d, C2), 69.7 (d, C4′), 73.1 (d, C3′), 76.3, 76.6 (d, $OCHPh_2$, C5′), 84.0 (d, C1′), 127.0, 127.3 (2×d, PhCH, C12), 127.8, 128.1, 128.5, 128.56 and 128.63 (5xd, 5xPhCH), 140.07 (s, PhC), 140.11 (s, PhC), 166.9 (s, C6′), 168.7 (s, C13), 169.27 (s, $\textit{C}OCH_3$), 169.31 (s, $\textit{C}OCH_3$), 170.1 (s, $\textit{C}OCH_3$), 175.2 (s, C29), 200.2 (s, C11); HRMS (*m*/*z*): $[M + H]^+$ calcd for 1012.5239; found: 1012.5236.

(3β,18β,20β)-3-*N*-{2-[Methyl (2,3,4-tri-*O*-acetyl-1-thio-β-D-glucopyranosyl)uronate]-ethyl-acetylamino}-11-oxo-olean-12-en-29-oic acid diphenylmethyl ester (9). A solution of amine **8** (2.11 g, 2.08 mmol, 1.0 equiv) in dry DCM (30 mL) was purged with Ar for 15 min and cooled to 0 °C. After the addition of triethylamine (2.0 mL, 14.6 mol, 7.0 equiv) and slow addition of Ac_2O (0.98 mL, 10.4 mmol, 5.0 equiv) the reaction mixture was stirred at 0 °C for 2 h and then at rt. Upon complete conversion the reaction mixture was diluted with EtOAc, washed two times with 10% AcOH, water, satd. aq $NaHCO_3$ and brine, dried ($MgSO_4$) and evaporated. The crude material was purified by MPLC (SiO_2: 114 g, Tol/EtOAc 2.5:1 → 1.5:1) to give pure **9** (2.10 g, 95.6%) as a white solid foam. R_f 0.27 (Tol/EtOAc 1:1); $[\alpha]_D^{20}$ +50.3 (*c* 0.8, $CHCl_3$); NMR analysis: Two rotamers in a ratio of 6:4 are observed. ^1H NMR ($CDCl_3$) δ 0.66, 0.67 (2×s, H28), 0.72–1.03 (m, 8H, H23, H24, H5, H16b), 1.03–1.20 (11H, H1b, H15b, H25, H26, H30), 1.20–1.93, (m, 13H, H2a, H6a, H6b, H7a, H7b, H15a, H19b, H21b, H22a, H22b, H27) 1.93–2.11 (m, 14H, 3×OAc, H2a, H21a, H16a, H18, H19a), 2.14 (s, 1.8H, NAc), 2.20 (s, 1.2H, NAc), 2.35 (s, 0.6H, H9), 2.38 (s, 0.4H, H9), 2.50–2.76, (m, 1H, SCH_2), 2.82–3.10 (m, 2H, H1, SCH_2), 3.18–3.32 (m, 1H, 0.6×H3, NCH_2) 3.36 (dd, *J* = 12.4 Hz, *J* = 3.0 Hz, 0.6H, H3), 3.40–3.76 (m, 1H, NCH_2), 3.74 (s, 1.8H, OCH_3), 3.74 (s, 1.2H, OCH_3), 4.06 (d, *J* = 9.9 Hz, 0.4H, H5′), 4.12 (d, *J* = 9.8 Hz, 0.6H, H5′), 4.47 (dd, *J* = 12.8 Hz, *J* = 3.0 Hz, 0.4H, H3), 4.55 (d, *J* = 9.9 Hz, 0.4H, H1′), 4.80 (d, *J* = 10.1 Hz, 0.6H, H1′), 5.00–5.09 (m, 1H, H2′), 5.05 (m, 1H, H2′), 5.17 (*app*. t, *J* =

9.7 Hz, 0.4H, H4′), 5.20 (*app*. t, J = 9.7 Hz, 1H, H4′), 5.28–5.34 (m, 1H, H3′), 5.51 (s, 0.4H, H12), 5.53 (s, 0.6H, H12), 6.93 (s, 1H, $OCHPh_2$), 7.26–740 (m, 10H, 10×PhH); ^{13}C NMR ($CDCl_3$) δ 16.5, 17.5, 17.7, 18.6, 18.8, 20.45, 20.50, 20.59, 20.63, 20.72, 22.0 (q, NCOCH_3), 23.1 (q, NCOCH_3), 23.27, 23.33, 23.9, 26.3, 26.4, 28.2, 28.3, 28.5, 28.7, 29.0, 30.3, 31.1, 31.7, 32.6, 37.0, 37.2, 37.4, 40.4, 40.79, 40.84, 41.0, 41.08, 41.16, 43.15, 43.18, 44.0, 45.2, 45.3, 45.5, 47.3, 47.97 (d, C18), 48.04 (d, C18), 52.8 (q, OCH_3), 53.0 (q, OCH_3), 55.8 (d, C5), 57.1 (d, C5), 59.9 (d, C3), 61.7 (d, C9), 66.7 (d, C3), 69.1, 69.2, 69.4 (3×d, 2×C4′, C2′), 69.7 (d, C2′), 72.6 (d, C3′), 73.1 (d, C3′), 76.0 (d, C5′), 76.2 (d, C5′), 76.6 (d, $OCHPh_2$), 83.6 (d, C1′), 84.3 (d, C1′), 126.9, 127.0, 127.2, 127.80, 127.83 and 128.09 (6 x d, 6 x PhCH), 128.12 (d, C12), 128.5 (d, C12), 128.6 (d, PhCH), 140.00 (s, PhC), 140.05 (s, PhC), 140.08 (s, PhC), 140.13 (s, PhC), 166.6 (s, C6′), 167.0 (s, C6′), 169.0, 169.1, 169.25, 169.29 (4×s, 2×C13, 2×O*C*OCH_3), 169.35 (s, O*C*OCH_3), 169.42 (s, O*C*OCH_3), 169.9 (s, O*C*OCH_3), 170.0 (s, O*C*OCH_3), 171.6 (s, NCOCH_3), 175.16 (s, C29), 175.22 (s, C29), 199.7 (s, C11), 200.1 (s, C11).

(3β,18β,20β)-3-*N*-{2-[Methyl (2,3,4-tri-*O*-acetyl-1-thio-β-D-glucopyranosyl)uronate]-ethylamino}-11-oxo-olean-12-en-29-oic acid (10). To a solution of ester **8** (400 mg, 0.395 mmol, 1.0 equiv) in dry DCM (20 mL) anisole (0.43 mL, 3.95 mmol, 10.0 equiv) was added and the reaction mixture was cooled to 0 °C. TFA (1.32 mL, 17.8 mmol, 45.0 equiv) was added dropwise, and the reaction mixture was allowed to come to rt and was stirred while being monitored by HPLC ($CHCl_3$/MeOH 9:1 + 0.5% AcOH). After 3 h all of the starting material was consumed. The solution was made neutral by the addition of satd. aq. $NaHCO_3$ (50 mL) at 0 °C (pH ~8). The phases were separated, the aqueous layer was extracted with DCM (4 x). The combined organic layers were washed with $NaHCO_3$, water and brine, dried (Na_2SO_4) and concentrated. The crude material was submitted to column chromatography (SiO_2: 20 g,

$CHCl_3$/MeOH 20:1 → 15:1) to give pure **10** (250 mg, 74.8%) as white solid foam. R_f 0.08 ($CHCl_3$/MeOH 9:1 + 0.1% AcOH); $[\alpha]_D^{20}$ +106.6 (*c* 1.0, $CHCl_3$); ^{1}H NMR ($CDCl_3$) δ 0.73 (m, 1H, H5), 0.82 (s, 3H, H28), 0.86 (s, 3H, H24), 0.85–0.95 (m, 1H, H1b), 0.95–1.05 (m, 1H, H16b), 1.07 (s, 3H, H23), 1.13 (s, 3H, H26), 1.14 (s, 3H, H25), 1.15–1.25 (m, 1H, H15b), 1.19 (s, 3H, H30), 1.30–1.52 (m, 5H, H6b, H7b, H21b, H22a, H22b), 1.37 (s, 3H, H27), 1.55–1.80 (m, 5H, H2a, H2b, H6a, H7a, H19b), 1.80–2.10 (m, 4H, H15a, H16a, H19a, H21a), 2.02 (bs, 6H, 2×OAc), 2.050 (s, 3H, OAc), 2.15–2.23 (m, 1H, H18), 2.25–2.35 (m, 1H, H3), 2.34 (s, 1H, H9), 2.80–3.05 (m, 4H, H1a, NCH_2, SCH_2), 3.25–3.35 (m, 1H, 1×NCH_2), 3.73 (s, 3H, OCH_3), 4.11 (d, *J* = 9.9 Hz, 1H, H5′), 4.66 (d, *J* = 10.0 Hz, 1H, H1′), 5.05 (*app*. t, *J* = 9.6 Hz, 1H, H2′), 5.19 (*app*. t, *J* = 9.8 Hz, 1H, H4′), 5.28 (*app*. t, *J* = 9.2 Hz, 1H, H3′), 5.68 (s, 1H, H12); ^{13}C NMR ($CDCl_3$) δ 16.21 (q, C25), 16.47 (q, C24), 17.71 (t, C6), 18.7 (q, C26), 20.5 (q, CO*C*H_3), 20.6 (q, CO*C*H_3), 20.7 (q, CO*C*H_3), 23.3 (t, C2), 23.4 (q, C27), 26.43 (t, C15), 26.50 (t, C16), 28.2 (q, C30), 28.5 (q, C23), 28.6 (q, C28), 29.6 (t, SCH_2), 31.1 (t, C21), 31.9 (s, C17), 32.8 (t, C7), 37.1 (s, C10), 37.8 (t, C22), 38.2 (s, C4), 39.4 (t, C1), 41.1 (t, C19), 43.2 (s, C20), 43.8, 45.4 (s, C8, C14), 47.0 (t, NCH_2), 48.3 (d, C18), 52.9 (q, OMe), 55.9 (d, C5), 61.7 (d, C9), 66.7 (d, C3), 69.3 (d, C4′), 69.5 (d, C2′), 73.0 (d, C3′), 76.0 (d, C5′), 84.0 (d, C1′), 128.5 (d, C12), 167.0 (s, C6′), 169.35, 169.43, 169.9 (s, C13, 2× *C*OCH_3) (s (s), 180.6 (s, C29), 200.0 (s, C11); HRMS (*m*/*z*): $[M + H]^+$ calcd for 846.4457; found, 846.4460.

(3β,18β,20β)-3-*N*-{2-[Methyl (1-thio-β-D-glucopyranosyl)uronate]ethylamino}-11-oxo-olean-12-en-29-oic acid (12). Peracetate **10** (0.260 g, 0.287 mmol, 1.00 equiv) was dissolved in dry MeOH and 0.1 M NaOMe was added at 0 °C to adjust the pH to ~10.5. The reaction mixture was stirred at 0 °C while being monitored by TLC ($CHCl_3$/MeOH 15:1 + 0.5% AcOH → 3:1 + 0.5% AcOH). Upon complete conversion, the reaction mixture was acidified (pH 6) by addition

of freshly washed Dowex 50W H^+ cation exchange resin. The resin was filtered off, washed with MeOH and the filtrate was concentrated to give pure **12** (200 mg, 89.4%) as a white solid. R_f 0.22 (EtOAc/MeOH 1:1 + 0.5% AcOH); $[\alpha]_D^{20}$ +97.9 (*c* 1.0, MeOH); ^{1}H NMR (CD_3OD) δ 0.83 (s, 3H, H28), 0.85–0.95 (m, 1H, H5), 0.92 (s, 3H, H24), 0.98–1.08 (m, 2H, H1b, H16b), 1.11 (bs, 6H, H23, H30), 1.16 (s, 3H, H26), 1.18 (s, 3H, H25), 1.20–1.41 (m, 3H, H15b, H21b, H22b), 1.41–1.56 (m, 3H, H6b, H7b, H22a), 1.42 (s, 3H, H27), 1.60–2.00 (m, 8H, H2a, H2b, H6a, H7a, H15a, H19a, H19b, H21a), 2.15 (td, *J* = 13.4 Hz, *J* = 4.1 Hz, 1H, H16a), 2.28 (dd, *J* = 13.0 Hz, *J* = 3.5 Hz, 1H, H18), 2.48 (s, 1H, H9), 2.70 (m, 1H, H3), 2.83 (m, 1H, H1a), 2.95–3.05 (m, 2H, SCH_2), 3.28 (m, 1H, H2′), 3.10–3.30 (m, 2H, NCH_2), 3.40 (*app*. t, *J* = 8.6 Hz, 1H, H3′), 3.53 (*app*. t, *J* = 9.0 Hz, 1H, H4′), 3.78 (s, 3H, OCH_3), 3.92 (d, *J* = 9.5 Hz, 1H, H5′), 4.54 (d, *J* = 9.2 Hz, 1H, H1′), 5.70 (s, 1H, H12); ^{13}C NMR (CD_3OD) δ 16.7 (q, C24), 16.8 (q, C25), 18.6 (t, C6), 19.3 (q, C26), 22.2 (t, C2), 23.8 (q, C27), 27.5 (t, C15), 27.7 (t, C16), 28.2 (t, SCH_2), 28.3 (q, C23), 29.28, 29.31 (2×q, C28, C30), 32.7 (t, C21), 33.0 (s, C17), 33.7 (t, C7), 38.2 (s, C10), 38.8 (s, C4), 39.4 (t, C22), 39.8 (t, C1), 43.3 (t, C19), 44.7 (s, C20), 45.8, 46.6 (s, C8, C14), 47.6 (t, NCH_2), 50.0 (s, C18), 53.0 (q, O*C*H_3), 56.5 (s, C5), 62.8 (s, C9), 67.5 (s, C3), 72.9 (s, C4′), 73.5 (s, C2′), 78.8 (s, C3′), 80.1 (s, C5′), 87.7 (s, C1′), 128.9 (s, C12), 171.1 (s, C6′), 173.6 (s, C13), 183.0 (s, C29), 202.2 (s, C11); HRMS (*m/z*): $[M + H]^+$ calcd for 720.4140; found, 720.4142.

(3β,18β,20β)-3-*N*-[2-(1-Thio-β-D-glucopyranosyluronic acid)ethylamino]-11-oxo-olean-12-en-29-oic acid (13). Methyl ester **12** (0.200 g, 0.277 mmol, 1.0 equiv) was dissolved in dry MeOH (3 mL) and 0.2 M NaOH in MeOH (6 mL) was added at 0 °C. The reaction mixture was stirred at rt under monitoring by TLC (samples were acidified with AcOH prior to elution in $CHCl_3$/MeOH 10:3). Upon complete conversion (3 h) the pH of the reaction mixture was

adjusted to pH 6–7 by adding Dowex 50W H^+ cation-exchange resin. The resin was removed by filtration, washed with MeOH and the filtrate was concentrated to give pure **13** (200 mg, >91%) as a white solid. R_f 0.12 (EtOAc/MeOH 1:1 + 0.5% AcOH); $[\alpha]_D^{20}$ +119.5 (*c* 1.0, MeOH); ^{1}H NMR (CD_3OD) δ 0.74 (s, 3H, H28), 0.85–0.95 (m, 2H, H5, H16b), 0.91 (s, 3H, H24), 1.01 (m, 1H, H1b), 1.02 (s, 3H, H30), 1.06 (s, 3H, H26), 1.09 (s, 3H, H25), 1.10 (s, 3H, H23), 1.10–1.30 (m, 3H, H15b, H21b, H22b), 1.34 (s, 3H, H27), 1.34–1.47 (m, 3H, H6b, H7b, H22a), 1.50–1.65 (m, 2H, H6a, H19b), 1.66–1.90 (m, 6H, H2a, H2b, H7a, H15a, H19a, H21a), 2.07 (td, *J* = 13.5 Hz, *J* = 4.3 Hz, 1H, H16a), 2.20 (dd, *J* = 13.1 Hz, *J* = 4.7 Hz, 1H, H18), 2.42 (s, 1H, H9), 2.78 (m, 1H, H1a), 2.90 (dd, *J* = 11.8 Hz, *J* = 4.7 Hz, 1H, H3), 2.95–3.10 (m, 2H, SCH_2), 3.12–3.17 (m, 1H, H2′), 3.28–3.40 (m, 2H, H3′, H4, NCH_2), 3.53–3.60 (m, 1H, H5′), 4.50 (d, *J* = 9.8 Hz, 1H, H1′), 5.59 (s, 1H, H12); ^{13}C NMR (CD_3OD) δ 16.7 (q, C24, C25), 18.5 (t, C6), 19.3 (q, C26), 20.9 (t, C2), 23.8 (q, C27), 27.2 (t, SCH_2), 27.5, 27.7 (2×t, C15, C16), 28.2 (q, C23), 29.3 (q, C28, C30), 32.7 (t, C7), 33.0 (s, C17), 33.6 (t, C21), 38.1 (s, C10), 38.8 (s, C4), 39.36 (t, C22), 39.44 (t, C1), 43.3 (t, C19), 44.7 (s, C20), 45.3 (t, NCH_2), 45.8, 46.6 (2×s, C14, C8), 50.0 (d, C18), 56.3 (d, C5), 62.6 (d, C9), 65.8 (d, C3), 73.4 (d, C4′), 74.2 (d, C2′), 78.8 (d, C5′), 79.2 (d, C3′), 86.8 (d, C1′), 128.8 (d, C12), 173.7 (s, C13), 176.2 (s, C6′), 183.1 (s, C29), 202.2 (s, C11); HRMS (*m*/*z*): $[M + H]^+$ calcd for 706.3983; found, 706.3984.

(3β,18β,20β)-3-*N*-{2-[Methyl (2,3,4-tri-*O*-acetyl-1-thio-β-D-glucopyranosyl)uronate]-ethyl-acetylamino}-11-oxo-olean-12-en-29-oic acid (11). To a cooled solution of ester **9** (2.00 g, 1.90 mmol, 1.0 equiv) in dry DCM (190 mL) first anisole (2.07 mL, 18.9 mmol, 10.0 equiv) and then slowly TFA (6.3 mL, 85.4 mmol, 45.0 equiv) was added. The reaction mixture was stirred at 0 °C for 1 h and then at rt while being monitored by TLC ($CHCl_3$/MeOH 15:1). After complete conversion (3 h), satd. aq. $NaHCO_3$ was added and the pH was adjusted with AcOH to ~4.5. The

phases were separated, the aqueous layer was extracted with DCM, and the combined organic layers were washed with brine, dried (Na_2SO_4) and concentrated. The crude material was purified by MPLC (SiO_2: 60 g, $CHCl_3$ → $CHCl_3$/MeOH 30:1) to give pure **11** (1.50 g, 89%) as a white solid. R_f 0.24 ($CHCl_3$/MeOH 9:1 + 0.1% AcOH); $[\alpha]_D^{20}$ +47.7 (*c* 0.9, $CHCl_3$); ^{1}H NMR ($CDCl_3$, a ratio of 5.8:4.2 was observed for two rotamers) δ 0.76–1.10 (m, 12H, H1b, H5, H16b, H23, H24, H28), 1.10–1.22 (m, 7H, H15b, H25, H26), 1.23 (s, 3H, H30), 1.25–1.73 (m, 12H, H2b, H6a, H6b, H7a, H7b, H19b, H21b, H22a, H22b, H27), 1.75–2.10 (14H, H2a, H15a, H19a, H21a, H16a, 3×OAc), 2.12–2.24 (m, 1H, 18H), 2.15 (s, 1.8H, NAc), 2.20 (s, 1.2H, NAc, 2.38 (s, 0.6H, H9), 2.40 (s, 0.4H, H9), 2.53–2.76 (m, 1.2H, SCH_2), 2.80–3.10 (m, ~2H, H1a, SCH_2), 3.22–3.32 (m, 0.6H, NCH_2), 3.37 (dd, *J* = 12.3 Hz, *J* = 3.0 Hz, 0.6H, H3), 3.42–3.76 (m, 1.4H, NCH_2, NCH_2), 3.73 (s, 1.8H, OCH_3), 3.74 (s, 1.2H, OCH_3), 4.07 (d, *J* = 9.9 Hz, 0.4H, H5′), 4.14 (d, *J* = 10.0 Hz, 0.6H, H5′), 4.46 (dd, *J* = 13.0 Hz, *J* = 3.2 Hz, 0.4H, H3), 4.55 (d, *J* = 10.0 Hz, 0.4H, H1′), 4.81 (d, *J* = 10.2 Hz, 0.6H, H1′), 5.00–5.07 (m, 1H, H2′), 5.17 (*app*. t, *J* = 9.7 Hz, 0.4H, H4′), 5.20 (*app*. t, *J* = 9.7 Hz, 0.6H, H4′), 5.308 (*app*. t, *J* = 9.4 Hz, 0.4H, H3′), 5.31 (*app*. t, *J* = 9.3 Hz, 0.6H, H3′), 5.70 (s, 0.4H, H12), 5.73 (s, 0.6H, H12); ^{13}C NMR ($CDCl_3$) δ 16.5 (q, C25), 17.5 (t, C6), 17.7 (t, C6), 18.6 (q, C26), 18.8 (q, C24), 20.4, 20.5, 20.6, 20.6 and 20.7 (5xq, 5xCO*C*H_3), 22.0 (q, NCO*C*H_3), 23.0 (q, NCO*C*H_3), 23.3 (t, C2), 23.4 (q, C27), 24.0 (t, C2), 26.4 (t, C15), 26.5 (t, C16), 28.4 (q, C23), 28.5 (q, C30), 28.7 (q, C28), 29.0 (t, SCH_2), 30.4 (t, SCH_2), 30.9 (t, C21), 31.9 (s, C17), 32.6 (t, C7), 37.1 (s, C10), 37.3 (s, C10), 37.7 (t, C22), 40.3, 40.8, 40.86, 40.92 (4×t, 2×C1, 2×C19), 41.2 (s, C4), 43.2 (s, C14), 43.3 (s, C14), 43.7 (s, C20), 45.3 (s, C8), 45.4 (s, C8), 45.6 (t, NCH_2), 47.4 (t, NCH_2), 48.2 (t, C18), 48.3 (t, C18), 52.8 (q, OCH_3), 53.0 (q, OCH_3), 55.8 (t, C5), 57.1 (t, C5), 60.1 (t, C3), 61.7 (t, C9), 66.8 (t, C3), 69.2, 69.3, 69.4, 69.8 (4×t, 2×C4′, 2×C2′), 72.7 (t, C3′), 73.1 (t, C3′), 76.0 (t, C5′), 76.3 (t, C5′), 83.6 (t, C1′), 84.3 (t, C1′), 128.5 (t, C12), 166.6 (s, C6′), 167.0 (s, C6′), 169.26, 169.31, 169.4, 169.6 (4×s, 2×C13,

$2\times OCOCH_3$), 169.9 (s, O*C*OCH$_3$), 170.0 (s, O*C*OCH$_3$), 171.8 (s, N*C*OCH$_3$), 171.9 (s, N*C*OCH$_3$), 180.7 (s, C29), 180.9 (s, C29), 200.0 (s, C11), 200.3 (s, C11); HRMS (*m*/*z*): $[M + H]^+$calcd for 888.4562; found, 888.4552.

(3β,18β,20β)-3-*N*-{2-[Methyl (1-thio-β-D-glucopyranosyl)uronate]-ethyl-acetylamino}-11-oxo-olean-12-en-29-oic acid (14). Peracetate **11** (1.57 g, 1.76 mmol, 1.0 equiv) was suspended in dry MeOH and 1 M NaOMe in MeOH (2.3 mL) was added at 0 °C. The suspension turned to a clear solution and the pH was adjusted to ~10. The reaction mixture was allowed to reach rt and was stirred under monitoring by TLC (EtOAc/MeOH 3:2 + 0.5% AcOH). Upon complete conversion the solution was treated as described for **12** to afford pure **14** (1.31 g, 97.4%) as a white solid. R_f 0.22 (EtOAc/MeOH 5:1 + 0.5% AcOH); $[\alpha]_D^{20}$ +39.9 (*c* 1.1, $CHCl_3$/MeOH 3:1); NMR analysis: Two rotamers in a ratio of 5.8:4.2 were observed. ^{1}H NMR ($CDCl_3$/CD_3OD 5:1) δ 0.78–1.12 (m, 12H, H1b, H5, H16b, H23, H24, H28), 1.13–1.33 (11H, H15b, H21b, H25, H26, H30), 1.33–1.77 (m, 11H, H2b, H6a, H6b, H7a, H7b, 19b, H22a, H22b, H27), 1.79–2.28 (m, 9H, H2a, H15a, H16a, H18, H19a, H21a, NAc), 2.43 (s, 1H, H9), 2.44 (s, 1H, H9), 2.55–3.14 (m, 3H, H1a, SCH_2), 3.27–3.53 (m, ~3H, H3*, H2′, H3′, NCH_2*), 3.53–3.77 (m, ~2.5H, NCH_2*, H4′), 3.80 (s, 1.8H, OCH_3), 3.81 (s, 1.2H, OCH_3), 3.88 (d, *J* = 6.6 Hz, 0.4H, H5′), 3.91 (d, *J* = 6.7 Hz, 0.6H, H5′), 4.42 (m, ~0.5H, H3), 4.48 (d, *J* = 9.9 Hz, ~0.5H, H1′), 4.51 (d, *J* = 9.6 Hz, ~0.5H, H1′), 5.66 (s, 0.4H, H12), 5.68 (s, 0.6H, H12); ^{13}C NMR ($CDCl_3$/CD_3OD 5:1) δ 16.1, 17.1, 17.3, 18.2, 18.4, 21.2, 22.5, 22.9, 23.0 (4×q, 2×NCO*C*H$_3$, 2×C27), 23.5, 26.0, 28.0, 28.1, 28.2, 28.4, 30.1, 30.6, 31.5, 32.2, 36.8, 36.9, 37.3, 40.0, 40.3, 40.5, 40.8, 43.0, 43.4, 45.1, 45.2, 45.6 (t, NCH_2), 47.2 (t, NCH_2), 47.9 (d, C18), 52.05 (q, OCH_3), 52.13 (q, OCH_3), 55.6 (d, C5), 56.6 (d, C5), 60.1 (d, C3), 61.4 (d, C9), 66.6 (d, C3), 70.9 (d, C4′), 71.1 (d, C4′), 71.8 (d, C2′), 71.9 (d, C2′), 77.2 (d, C3′), 78.3 (d, C5′), 85.8 (d, C1′), 86.7 (d, C1′), 127.6 (d, C12), 168.9 (s,

C6′), 169.2 (s, C6′), 170.9 (s, C13), 172.34 (s, N*C*OCH$_3$), 172.39 (s, N*C*OCH$_3$), 178.96 (s, C29), 179.01 (s, C29), 200.6 (s, C11), 201.0 (s, C11); HRMS (*m*/*z*): $[M + H]^+$ calcd for 762.4245; found, 762.4256.

(3β,18β,20β)-3-*N*-[2-(1-Thio-β-D-glucopyranosyluronic acid)-ethyl-acetylamino]-11-oxo-olean-12-en-29-oic acid (15). Methyl ester **14** (1.31 g, 1.72 mmol, 1.0 equiv) was dissolved in 0.2 M NaOH in dry MeOH (91 mL) and stirred under monitoring by TLC (EtOAc/MeOH 3:2 + 0.5% AcOH). Upon complete conversion the solution was processed as described for **13**. Lyophilization from 1,4-dioxane furnished **15** (1.34 g, quantitative) as a colorless solid. R_f 0.07 (EtOAc/MeOH 3:2 + 0.5% AcOH); $[\alpha]_D^{20}$ +38.0 (*c* 9.7, MeOH/$CHCl_3$ 5:1); NMR analysis: Two rotamers in a ratio of 5.6:4.4 were observed. ^{1}H NMR (CD_3OD) δ 0.78–1.10 (m, 11H, H5, H16b, H23, H24, H28), 1.10–1.33 (11H, H1b, H15b, H25, H26, H30), 1.33–1.66 (m, 10H, H2b, H6a, H6b, H7b, H21b, H22a, H22b, H27), 1.66–2.05 (m, 5H, H7a, H15a, H19a, 19b, H21a), 2.06–2.27 (m, 6H, H2a, H16a, H18, NAc), 2.51 (s, 0.4H, H9), 2.53 (s, 0.6H, H9), 2.60–3.09 (m, 3H, H1a, SCH_2), 3.24–3.29 (m, 1H, H2′), 3.35–3.46 (m, ~1.5H, H3′, NC*H*$_2$), 3.48–3.78 (m, ~3H, H3, H4′, NC*H*$_2$), 3.83 (d, *J* = 9.7 Hz, 1H, H5′), 3.84 (d, *J* = 9.8 Hz, 1H, H5′), 4.43 (dd, *J* = 13.0 Hz, *J* = 3.1 Hz, 0.4H, H3), 4.53 (d, *J* = 9.8 Hz, 1H, H1′), 4.54 (d, *J* = 9.6 Hz, 1H, H1′), 5.59 (s, 0.6H, H12), 5.60 (s, 0.4H, H12); ^{13}C NMR (CD_3OD) δ 17.19, 17.2, 18.7, 18.8, 19.2, 19.5, 19.6, 22.1 (q, NCO*C*H$_3$), 23.1 (q, NCO*C*H$_3$), 23.8 (q, C27), 23.9, 24.4, 24.8, 27.4, 27.6, 28.8, 29.0, 29.2, 29.8 (t, SCH_2), 31.3 (t, SCH_2), 32.0, 33.0, 33.6, 33.7, 38.4, 38.5, 39.0, 41.5, 41.7, 41.9, 42.2, 42.4, 44.6, 44.9, 46.65, 46.69, 47.5 (t, NCH_2), 49.9 (d, C18), 57.5 (d, C5), 57.7 (d, C5), 61.9 (d, C3), 63.0 (d, C9), 63.1 (d, C9), 68.0 (d, C3), 73.0 (d, C4′), 73.9 (d, C2′), 74.0 (d, C2′), 78.85 (d, C3′), 78.94 (d, C3′), 80.1 (d, C5′), 87.2 (d, C1′), 88.4 (d, C1′), 128.9 (d, C12), 172.5 (s, C13), 172.8 (s,

C13), 174.47 (s, N*C*OCH_3), 174.52 (s, N*C*OCH_3), 180.3 (s, C29), 202.4 (s, C11), 202.5 (s, C11); HRMS (*m*/*z*): $[M + H]^+$ calcd for 748.4089; found, 748.4097.

(3β,18β,20β)-3-*N*-{2-[Methyl (2,3,4-tri-*O*-acetyl-1-thio-β-D-glucopyranosyl)uronate]-ethyl-(methylsuccinyl)amino}-11-oxo-olean-12-en-29-oic acid (17). DIPEA (0.29 mL, 1.73 mmol, 5.00 equiv) was added to a solution of amine **8** (0.350 g, 0.346 mmol, 1.00 equiv) in dry DCM (10 mL) and the reaction mixture was cooled to 0 °C. Methyl succinoyl chloride (0.156 g, 1.04 mmol, 3.0 equiv) was added gradually at 0 °C within 5 min and the reaction mixture was stirred at 0 °C under monitoring by HPLC. Upon complete conversion (1.5 h), MeOH (2 mL) was added and the reaction mixture was allowed to reach rt. The reaction mixture was distributed between satd. NH_4Cl and EtOAc (40 mL) and the phases were separated. The aqueous layer was extracted with EtOAc and the combined organic layers were washed with satd. NH_4Cl and brine, dried (Na_2SO_4) and concentrated. The crude material was purified by column chromatography (SiO_2: 17 g, Hex/EtOAc 3:1 → 3:2) to give the *N*-acylated intermediate (379 mg, 97.3%) as a slightly yellowish solid foam. Analytical data of the intermediate ester: R_f 0.34 (EtOAc/Hex 2:1). An aliquot of the intermediate diphenylmethyl ester (0.350 g, 0.311 mmol, 1.00 equiv) was dissolved in dry DCM (15 mL) and first anisole (0.34 mL, 3.1 mmol, 10.0 equiv) and then TFA (1.04 mL, 14.0 mmol, 45 equiv) was added gradually at 0 °C. The reaction mixture was stirred under monitoring by TLC (Hex/EtOAc 1:2 + 0.5% AcOH) at 0 °C. Upon complete conversion (3 h) the reaction mixture was allowed to reach rt and was diluted with EtOAc. The EtOAc phase was washed with water and brine, dried (Na_2SO_4) and concentrated. The crude material was purified by column chromatography (SiO_2: 45 g, Hex/EtOAc 2:1 → 1:2 + 0.1% AcOH) to give pure **17** (248 mg, 83%) as a white solid. R_f = 0.46 ($CHCl_3$/MeOH 9:1 + 0.1% AcOH); $[\alpha]_D^{20}$ +38.3 (*c* 0.3, $CHCl_3$); NMR analysis: Two rotamers in a ratio of 6:4 are observed. ^1H NMR

($CDCl_3$) δ 0.71–1.09 (m, 12H, H1b, H5, H16b, H23, H24, H28), 1.10–2.30 (m, 37H, H2a, H2b, H6a, H6b, H7a, H7b, H15a, H15b, H16a, 18H, H19a, H19b, H21a, H21b, H22a, H22b, H25, H26, H27, H30, 3×OAc), 2.36 (s, 0.4H, H9), 2.40 (s, 0.6H, H9), 2.40–3.00 (m, 7H, SCH_2, 2×$COCH_2$, H1a), 3.21–3.74 (m, 2.6H, NCH_2, H3), 3.69, 3.70 (2×s, 3H, OCH_3), 3.74 (bs, 3H, OCH_3), 4.10 (d, J = 9.8 Hz, 0.4H, H5′), 4.16 (d, J = 9.8 Hz, 0.6H, H5′), 4.45 (dd, J = 13.0 Hz, J = 3.0 Hz, 0.4H, H3), 4.60 (d, J = 9.9 Hz, 0.4H, H1′), 4.81 (d, J = 10.2 Hz, 0.6H, H1′), 5.01 (*app*. t, J = 9.6 Hz, 0.6H, H2′), 5.11 (*app*. t, J = 9.6 Hz, 0.6H, H2′), 5.18 (*app*. t, J = 9.7 Hz, 0.4H, H4′), 5.19 (*app*. t, J = 9.6 Hz, 0.4H, H4′), 5.31 (*app*. t, J = 9.3 Hz, 1H, H3′), 5.31 (app. t, J = 9.3 Hz, 1H, H3′), 5.67 (s, 0.4H, H12), 5.70 (s, 0.6H, H12); ^{13}C NMR ($CDCl_3$) δ 16.4, 17.45, 17.7, 18.5, 18.8, 20.3 (q, CO*C*H_3), 20.4 (q, CO*C*H_3), 20.5 (q, CO*C*H_3), 20.6 (q, CO*C*H_3), 23.2, 23.3, 23.8, 26.3, 28.34, 28.4, 28.9, 29.2, 29.45, 31.0, 31.8, 32.5, 37, 37.2, 37.7, 40.3, 40.6, 40.8, 41.0 (t, C1), 43.2, 43.6, 45.3, 45.4, 45.7 (t, NCH_2), 46.6 (t, NCH_2), 48.2 (d, C18), 51.7 (q, OCH_3), 52.8 (q, OCH_3), 52.9 (q, OCH_3), 55.9 (d, C5), 57.0 (d, C5), 60.4 (d, C3), 61.7 (d, C9), 65.4 (d, C3), 69.2 (d, C2′), 69.4 (d, C4′), 69.8 (d, C2′), 72.7 (d, C3′), 73.1 (d, C3′), 75.9 (d, C5′), 76.1 (d, C5′), 83.5 (d, C1′), 84.0 (d, C1′), 128.3 (d, C12), 166.7 (s, C6′), 167.1 (s, C6′), 169.3, 169.4, 169.5, 169.9 (4×s, 2×C13, 2×CO), 169.9 (s, CO), 170.0 (s, CO), 170.1 (s, CO), 172.4 (s, CO), 172.7 (s, CO), 173.6 (s, CO), 173.8 (s, CO), 179.3 (s, C29), 200.2 (s, C11), 200.5 (s, C11); HRMS (*m*/*z*): $[M + H]^+$ calcd for 960.4774; found, 960.4776.

(3β,18β,20β)-3-*N*-{2-[Methyl (1-thio-β-D-gluco-pyranosyl)uronate]-ethyl-[(methylsuccinyl)amino]}-11-oxo-olean-12-en-29-oic acid (18). Peracetate **17** (70 mg, 0.073 mmol, 1.00 equiv) was dissolved in dry MeOH (2.5 mL) and 0.1 M NaOMe was added at 0 °C. The pH was adjusted to 10 and the reaction mixture was stirred at 0 °C under monitoring by TLC (EtOAc/Hex 2:1, DCM/MeOH 10:1 + 0.5% AcOH) and HPLC for 2.5 hours. Upon

complete conversion, the reaction mixture was acidified with Dowex 50W H^+ cation-exchange resin (pH 4–5) and filtered. The filtrate was concentrated and applied onto SiO_2 (200 mg) and purified by column chromatography (SiO_2: 8 g, DCM/MeOH 20:1 + 0.1% AcOH $\rightarrow$ 15:1 + 0.1% AcOH) to give pure **18** (60 mg, 98.7%) as a white solid after coevaporation from toluene. R_f 0.44 (EtOAc/MeOH 5:1 + 0.5% AcOH); $[\alpha]_D^{20}$ +37.4 (*c* 0.5, $CHCl_3$/MeOH 5:1); NMR analysis: Two rotamers in a ratio of 5.3:4.7 were observed. ^{1}H NMR ($CDCl_3$/CD_3OD 5:1) δ 0.73–0.99 (m, 10H, H5, H23, H24, H28), 0.98–1.24 (12H H1b, H15b, H16b, H25, H26, H30), 1.25–1.76 (m, 12H, H2b, H6a, H6b, H7a, H7b, 19b, H21b, H22a, H22b, H27), 1.77–2.28 (m, 6H, H2a, H15a, H16a, H18, H19a, H21a), 2.42 (s, 1H, H9), 2.55–3.08 (m, 7H, H1a, 2xCOCH$_2$, SCH$_2$), 3.27–3.61 (m, ~3H, H3, H2′, H3′, NCH$_2$*), 3.62–3.72 (m, ~5H, NCH$_2$*, H4′), 3.68 (s, 1.2H, OCH$_3$), 3.69 (s, 1.8H, OCH$_3$), 3.79 (s, 3H, OCH$_3$), 3.89 (d, *J* = 9.7 Hz, 0.4H, H5′), 3.91 (d, *J* = 9.8 Hz, 0.6H, H5′), 4.42 (dd, *J* = 13.0 Hz, *J* = 3.2 Hz, 0.4H, H3), 4.48 (d, *J* = 9.4 Hz, 0.4H, H1′), 4.50 (d, *J* = 9.5 Hz, 0.6H, H1′), 5.66 (s, 0.4H, H12), 5.68 (s, 0.6H, H1); ^{13}C NMR ($CHCl_3$/CD_3OD 5:1) δ 16.20, 16.24, 17.3, 17.5, 18.37, 18.40, 18.6, 23.0, 23.1, 23.6, 26.2, 26.3, 28.2, 28.3, 28.4, 29.1, 29.3, 29.9, 30.9, 31.7, 32.4, 36.9, 37.1, 37.5, 40.2, 40.5, 40.7, 40.9, 41.0, 43.2, 43.6, 45.2, 45.3, 45.9 (t, NCH$_2$), 46.5 (t, NCH$_2$), 48.2 (d, 18), 51.6 (q, OCH$_3$), 52.26 (q, C6′), 52.32 (q, C6′), 55.8 (d, C5), 56.8 (d, C5), 60.4 (d, C3), 61.6 (d, C9), 65.4 (d, C3), 71.0 (d, C4′), 71.2 (d, C4′), 71.85 (d, C2′), 71.94 (d, C2′), 77.2 (d, C3′), 77.3 (d, C3′), 78.3 (d, C5′), 78.4 (d, C5′), 85.7 (d, C1′), 86.7 (d, C1′), 127.9 (d, C12), 169.1 (s, C6′), 169.3 (s, C6′), 170.8 (s, C13), 172.7 (s, *C*OCH$_3$), 172.8 (s, *C*OCH$_3$), 173.7 (s, *C*OCH$_3$), 173.8 (s, *C*OCH$_3$), 179.6 (s, C29), 200.6 (s, C11), 200.9 (s, C11); HRMS (*m*/*z*): $[M + H]^+$ calcd for 834.4457; found, 834.4459.

(3β,18β,20β)-3-*N*-{2-[1-Thio-β-D-glucopyranosyluronic acid]-ethyl-[succinylamino]}-11-oxo-olean-12-en-29-oic acid (19). Dimethyl ester **18** (60 mg, 0.072 mmol, 1.00 equiv) was dissolved in 0.2 M NaOH at rt, water (0.4 mL) was added and the reaction mixture was stirred at rt overnight. According to HPLC complete conversion from the starting material to target compound was achieved. The pH of the reaction mixture was adjusted to ~8 by adding Dowex 50W H^+ cation-exchange resin. The suspension was filtered, washed and the filtrate was concentrated. The residue was taken up in a mixture of dioxane:water 1:1 (3 mL) and lyophilized to yield pure **19** (62 mg, 98.8%) as a colorless solid. R_f 0.04 (EtOAc/MeOH 3:2 + 0.5% AcOH); $[\alpha]_D^{20}$ +33.9 (*c* 0.6, MeOH); NMR analysis: Two rotamers in a ratio of 5.4:4.6 were observed. ^{1}H NMR (CD_3OD) δ 0.75–1.36 (m, 24H, H1b, H5, H15b, H16b, H22a, H22b, H23, H24, H25, H26, H28, H30), 1.37–1.76 (m, 16H, H2a, H2b, H6a, H6b, H7a, H7b, H15a, H16a, H18, 19b, H19a, H21a, H21b, H27), 2.26–3.22 (m, 9H, H1a, H9, H18, 2×$COCH_2$, SCH_2), 3.20–3.53 (m, 4H, H2′, H3′, H4′, NCH_2), 3.55–3.75 (m, 2.6H, H5′, NCH_2, H3), 4.37–4.47 (m, 0.4H, H3), 4.45 (d, *J* = 9.7 Hz, 0.6H, H1′), 4.50 (d, *J* = 9.6 Hz, 0.4H, H1′), 5.73 (s, 0.6H, H12), 5.74 (s, 0.4H, H12); ^{13}C NMR (CD_3OD) δ 17.1, 17.2, 18.7, 18.8, 19.2, 19.5, 19.7, 23.7, 23.8, 24.4, 24.8, 27.6, 29.0, 29.3, 29.36, 29.43, 29.6, 31.5, 31.7, 32.4, 33.0, 33.1, 33.7, 33.8, 34.7, 38.4, 38.5, 39.6, 41.5, 41.95, 42.00, 43.9, 44.6, 44.7, 46.4, 46.7, 46.9 (t, NCH_2), 47.9 (t, NCH_2), 50.0 (d, C18), 57.6 (d, C5), 57.8 (d, C5), 61.9 (d, C3), 62.9 (d, C9), 63.0 (d, C9), 66.9 (d, C3), 73.4 (d, C4′), 73.8 (d, C2′), 74.0 (d, C2′), 79.2 (d, C3′), 80.0 (d, C5′), 87.0 (d, C1′), 87.6 (d, C1′), 128.7 (d, C12), 174.4 (s, C13), 174.4 (s, C13), 176.4 (s, C6′), 176.5 (s, C6′), 176.7 (s, *C*OCH_2), 181.2 (s, *C*OCH_2), 181.2 (s, *C*OCH_2), 185.0 (s, C29), 202.9 (s, C11), 203.0 (s, C11); HRMS (*m*/*z*): $[M + H]^+$ calcd for 806.4144; found, 806.4151.

(3α,3β,18β,20β)-1-{2-[Methyl (2,3,4-tri-*O*-acetyl-1-thio-β-D-glucopyranosyl)uronate] ethylthio}-11-oxo-olean-2,12-dien-29-oic acid (21). A solution K_2CO_3 (90 mg, 0.66 mmol) in water (0.6 mL) was added to a solution of iodide **6** (125 mg, 0.25 mmol) and thiol **20** (80 mg, 0.165 mmol) in acetone (3 mL). The reaction mixture was stirred at rt overnight. The solution was diluted with EtOAc, washed with ice-cold 0.1 M HCl and water, dried over cotton and concentrated. The crude material was purified by column chromatography (Hex/EtOAc 3:2) to afford **21** (85 mg, 60%) as a yellow syrup. ^{1}H NMR ($CDCl_3$, 400 MHz) δ 0.84 (s, 3H, H28), 0.89, 1.00 (2×s, 3H, H24), 1.00–1.08 (m, 1H, H16b), 1.07, 1.12 (2×s, 3H, H23), 1.14 (s, 3H, H26), 1.15–1.78 (m, 10H, H5, H6a, H6b, H7a, H7b, H15b, H19b, H21b, H22a, H22b), 1.23 (s, 3H, H29), 1.28, 1.31 (2×s, 3H, H25), 1.38, 1.39 (2×s, 3H, H27), 1.78–2.12 (m, 4H, H15a, H16a, H19a, H21a), 2.02, 2.05, 2.06 (3×s, 9H, OAc), 2.15–2.24 (m, 1H, H18), 2.56, 2.58 (2×s, 1H, H9), 2.76–3.00 (m, 4.5H, H3, α-isomer), 2×SCH_2), 3.08 (*app*. t, *J* = 2.3 Hz, 0.5H, H3, β-isomer), 3.75 (s, 3H, OMe), 4.03 (d, *J* = 9.6 Hz, 0.5H, H5′), 4.04 (d, *J* = 9.6 Hz, 0.5H, H5′), 4.58 (d, *J* = 10.0 Hz, 0.5H, H1′), 4.58 (d, *J* = 10.0 Hz, 0.5H, H1′), 5.04 (dd, *J* = 9.7 Hz, *J* = 9.3 Hz, 1H, H2′), 5.21 (app. t, *J* = 9.3 Hz, 0.5H, H4′), 5.21 (*app*. t, *J* = 9.3 Hz, 0.5H, H4′), 5.27 (*app*. t, *J* = 9.1 Hz, 0.5H, H3′), 5.27 (*app*. t, *J* = 9.2 Hz, 0.5H, H3′), 5.40 (dd, *J* = 10.4 Hz, *J* = 1.9 Hz, 1H, H2, β-isomer), 5.54 (dd, *J* = 10.3 Hz, *J* = 5.2 Hz, 1H, H2, α-isomer), 5.71, 5.72 (2×s, 1H, H12), 6.62 (d, *J* = 10.4 Hz, 1H, H1, α-isomer), 6.67 (dd, *J* = 10.4 Hz, *J* = 2.6 Hz, 1H, H1, β-isomer); ^{13}C NMR ($CDCl_3$/MeOD, 100 MHz) δ 16.6, 17.3 (2×t, C6), 18.2 (q,C25), 19.0, 19.2, 19.4 (3×q, C24 β-isomer, C25, C26), 20.1, 20.2, 20.4 (3×q, 3×OAc), 23.2 (q, C27), 25.1 (q, C24, α-isomer), 26.1, 26.2 (2×t, C16), 28.1, 28.2, 28.5, 28.7 (4×q, C23, C28, C30), 30.4, 30.8 (2×t, C21), 31.6 (s, C17), 32.0, 32.9 (2×t, C7, SCH_2), 33.8 (t, SCH_2), 36.6, 36.8 (2×s, C4), 37.5 (t, C22), 37.9, 38.2 (2×s, C10), 40.9 (t, C19), 43.3, 43.5 (2×s, C14, C20), 45.5, 45.6 (2×s, C8), 48.3, 48.4 (2×d, C5, C18),

52.7 (q, OMe), 54.6, 55.2 (2×d, C3, α-isomer), C5), 56.1 (d, C3, β-isomer), 58.5 (d, C9), 69.2 (d, C4′), 69.4, 69.5 (2×d, C2′), 72.9 (d, C3′), 75.9 (d, C5′), 83.6, 83.8 (2×d, C1′), 122.8 (d, C2, α-isomer), 123.8 (d, C2, β-isomer), 127.6, 127.7 (2×d, C12), 138.6, 138.8 (2×d, C1), 166.9 (s, C6′), 169.5, 170.1 (2×s, 3×$COCH_3$), 171.0, 171.1 (2×s, C13), 179.1 (s, C29), 200.6, 200.8 (2×s, C11); HRMS (*m*/*z*): $[M + H]^+$ calcd for 859.3766; found, 859.3763.

(3α,3β,18β,20β)-1-{2-[Methyl (1-thio-β-D-gluco-pyranosyl)uronate]ethylthio}-11-oxo-olean-2,12-en-29-oic acid (22). Peracetate **21** (70 mg, 0.083 mmol) was dissolved in dry MeOH (3.5 mL) and ~0.8 M NaOMe (~150 µL) was added at rt to adjust the pH to ~11. The reaction mixture was stirred at rt under monitoring by TLC (EtOAc/MeOH 9:1). After 1 h complete consumption of starting material was observed and the reaction mixture was acidified with Dowex 50W H^+cation-exchange resin (pH 4–5) and filtered. The filtrate was concentrated and the crude material was purified by column chromatography (DCM/MeOH 19:1 → 9:1) to give pure **22** (43 mg, 71%) as a white solid. R_f 0.17 (DCM/MeOH 8:1); ^{1}H NMR (CD_3OD, 400 MHz) δ 0.84 (s, 3H, H28), 0.87 (s, 1.5H, H24 β-isomer), 1.01 (s, 1.5H, H24 α-isomer), 1.00–1.08 (m, 1H, H16b), 1.08 (s, 1.5H, H23), 1.14 (s, 1.5H, H23), 1.16 (s, 3H, H26), 1.17,1.18 (2×s, 3H, H30), 1.20–1.33 (m, 1.5H, H15b, H5′), 1.27, 1.30 (2×s, 3H, H25), 1.33–1.63 (m, 5.5H, H5′, H6b, H7b, H21b, H22a, H22b), 1.42 (s, 3H, H27), 1.63–2.02 (m, 5H, H6a, H7a, H15a, H19, H21a), 1.71 (app. t, *J* = 13.4 Hz, 1H, H19), 2.12–2.20 (m, 1H, H16a), 2.20 (td, *J* = 13.5 Hz, *J* = 3.8 Hz, 1H, H18), 2.64, 2.67 (2×s, 1H, H9), 2.76–2.95 (m, 4H, 2×SCH_2), 3.03 (dd, *J* = 5.3 Hz, *J* = 1.0 Hz, 1H, H3, δ-isomer), 3.12 (app. t, *J* = 2.3 Hz, 1H, H3, β-isomer), 3.23 (dd, *J* = 9.7 Hz, *J* = 8.8 Hz, 1H, H2′), 3.24 (dd, *J* = 9.7 Hz, *J* = 8.8 Hz, 1H, H2′), 3.37 (*app*. t, *J* = 8.5 Hz, 1H, H3′), 3.54 (*app*. t, *J* = 9.5 Hz, 1H, H4′), 3.546 (*app*. t, *J* = 9.5 Hz, 1H, H4′), 3.76 (s, 3H, OMe, β-isomer), 3.78 (s,

3H, OMe, α-isomer), 3.85 (d, J = 9.8 Hz, 1H, H5′), 3.85 (d, J = 9.8 Hz, 1H, H5′), 4.48 (d, J = 9.8 Hz, 1H, H1′), 4.49 (d, J = 9.8 Hz, 1H, H1′), 5.45 (dd, J = 10.5 Hz, 2.0 Hz, 1H, H2, β-isomer), 5.55 (dd, J = 10.2 Hz, J = 5.2 Hz, 1H, H2, α-isomer), 5.61, (s, 1H, H12), 6.54–6.49 (m, 1H, H1); ^{13}C NMR (CD_3OD, 100 MHz) δ 17.84, 18.60 (2×t, C6), 19.23, (q, C25), 19.88, 20.06, 20.13, 20.20 (4×q, C26, C24 β-isomer), 24.03, 24.08 (2×q, C27), 25.92 (q, C24 α-isomer), 27.34, 27.54 (2×t, C15, C16), 28.77, 29.20, 29.29 (3×q, C23, C28, C30), 31.77, 31.99 (2×t, C21), 32.98 (s, C17), 33.94, 34.12, 34.20 (3×t, C7, SCH_2), 35.34 (t, $CSCH_2$), 37.78, 38.10 (2×s, C4), 39.01 (t, C22), 39.41, 39.69 (2×s, C10), 42.26, 42.33 (2×t, C19), 44.78, 44.91 (2×s, C14, C20), 46.96, 49.79 (2×s, C8), 49.79, 50.00 (2×d, C18), 50.03 (d, C5), 52.86 (q, OMe, β-isomer), 52.89 (q, OMe, α-isomer), 55.93 (d, C5), 56.35 (d, C3, α-isomer), 57.46 (d, C3, β-isomer), 59.98, 60.11 (2×d, C9), 72.90 (d, C4′), 73.99 (d, C2′), 78.78 (d, C3′), 80.27, 80.31 (2×d, C5′), 87.97, 88.04 (2×d, C1′), 124.48 (d, C2, α-isomer), 126.02 (d, C2, β-isomer), 128.62, 128.68 (2×d, C12), 139.37, 139.42 (2×d, C1), 170.85, 170.89 (2×s, C6′), 173.32, 173.40 (2×s, C13), 180.36, 180.41 (2×s, C29), 202.31, 202.41 (2×s, C11); HRMS (m/z): $[M + H]^+$ calcd for 735.3595; found, 735.3599.

(3α,3β,18β,20β)-1-[2-(1-Thio-β-D-glucopyranosyluronic acid)ethylthio]-11-oxo-olean-2,12-en-29-oic acid (23). Methylester **22** (28 mg, 0.038 mmol) was dissolved in dry MeOH (1 mL). NaOH in dry MeOH (0.2 M, 1.1 mL) and water (200 µL) were added, and the reaction mixture was stirred at rt for 30 min. According to HPLC (after acidic workup of an aliquot with AcOH) and TLC (EtOAc/MeOH 3:2) complete conversion to one polar compound was achieved. The reaction mixture was acidified with Dowex 50W H^+cation exchange resin to pH 4–5. Filtration and concentration of the filtrate gave **23** (26 mg, 95%) as a white solid (according to NMR

contaminated with a small impurity). R_f 0.19 (tailing, EtOAc/MeOH 3:2); ^{1}H NMR (CD_3OD, 400 MHz) δ 0.84 (s, 3H, H28), 0.87 (s, 1.5H, H24 β-isomer), 1.01 (s, 1.5H, H24 α-isomer), 1.00–1.08 (m, 1H, H16b), 1.09 (s, 1.5H, H23), 1.14 (s, 1.5H, H23), 1.15 (s, 3H, H26), 1.17, 1.18 (2×s, 3H, H30), 1.20–1.33 (m, 1.5H, H15b, H5*), 1.27, 1.30 (2×s, 3H, H25), 1.33–1.63 (m, 5.5H, H5*, H6b, H7b, H21b, H22a, H22b), 1.42 (s, 3H, H27), 1.63–2.02 (m, 5H, H6a, H7a, H15a, H19, H21a), 1.71 (*app*. t, J = 13.4 Hz, 1H, H19), 2.12–2.20 (m, 1H, H16a), 2.20 (td, J = 13.3 Hz, J = 4.1 Hz, 1H, H18), 2.63, 2.66 (2×s, 1H, H9), 2.76–2.94 (m, 1H, SCH_2), 3.03 (dd, J = 5.2 Hz, J = 1.0 Hz, 1H, H3, α-isomer), 3.13 (*app*. t, J = 2.3 Hz, 1H, H3, β-isomer), 3.235 (dd, J = 9.7 Hz, J = 8.8 Hz, 1H, H2′), 3.25 (dd, J = 9.7 Hz, J = 8.8 Hz, 1H, H2′), 3.38 (*app*. t, J = 8.9 Hz, 1H, H3′), 3.53 (*app*. t, J = 9.4 Hz, 1H, H4′), 3.77 (d, J = 9.8 Hz, 1H, H5′), 3.775 (d, J = 9.8 Hz, 1H, H5′), 4.48 (d, J = 9.7 Hz, 1H, H1′), 4.48 (d, J = 9.7 Hz, 1H, H1′), 5.47 (dd, J = 10.5 Hz, 2.0 Hz, 1H, H2, β-isomer), 5.56 (dd, J = 10.2 Hz, 5.2 Hz, 1H, H2, α-isomer), 5.61 (s, 1H, H12), 6.53–6.58 (m, 1H, H1); ^{13}C NMR (CD_3OD, 100 MHz) δ 17.8, 18.6 (2×t, C6), 19.2 (q, C25), 19.9, 20.05, 20.12, 20.2 (4×q, C25, C26, C24 β-isomer), 24.0, 24.1 (2×q, C27), 25.9 (q, C24 α-isomer), 27.3, 27.5 (2×t, C15, C16), 28.8, 29.2, 29.3 (3×q, C23, C28, C30), 31.7, 32.0 (2×t, C21), 33.0 (s, C17), 34.0, 34.1, 34.2 (t, C7, SCH_2), 35.4 (t, SCH_2), 37.8, 38.1 (2×s, C4), 39.0 (t, C22), 39.4, 39.7 (2×s, C10), 42.25, 42.32 (2×t, C19), 44.78, 44.81, 44.9 (3×s, C14), 46.98, 47.04 (2×s, C8), 49.8, 50.0, 50.1 (2×d, C5, C18), 55.9 (d, C5), 56.5 (d, C3, α-isomer), 57.5 (d, C3, β-isomer), 60.0, 60.1 (2×d, C9), 73.0 (d, C4′), 74.05, 74.07 (2×d, C2′), 79.0 (d, C3′), 80.2 (d, C5′), 87.8 (d, C1′), 124.6 (d, C2, α-isomer), 126.1 (d, C2, β-isomer), 128.6, 128.7 (2×d, C12), 139.3, 139.4 (2×d, C1), 172.7 (s, C6′), 173.3, 173.4 (2×s, C13), 180.38, 180.43 (2×s, C29), 202.4, 202.5 (s, C11); HRMS (*m*/*z*): $[M + H]^+$ calcd for 721.3452; found, 721.3450.

Biology

The influenza A virus (IAV) was obtained from American Tissue Culture Collection (A/Aichi/2/68 (H3N2); VR-547). Virus stocks were prepared by propagation of virus on Mardin-Darby canine kidney (MDCK; ATCC CCL-34) cells and infectious titers of virus stocks were determined by the 50% tissue culture infective dose (TCID50) analysis as described in [5]. MDCK cells were seeded in 96-well plates at 2×10^4 cells/well using DMEM/Ham's F-12 (1:1) medium containing 10% foetal bovine serum (FBS), 2 mM L-glutamine and 1% antibiotic–antimycotic solution (penicillin, 10.000 units/mL; streptomycin, sulfate 10 mg/mL; Amphotericin B, 25 µg/ml) (all from PAA). Until infection the cells were incubated for 5 h at 37 °C/5.0% CO_2 to form a ~80% confluent monolayer on the bottom of the well. Test compounds were dissolved in DMSO and dilution series were prepared in infection medium (DMEM/Ham's F-12 (1:1) containing 5 µg/mL trypsin (Sigma T4945), and 1% antibiotic–antimycotic solution) resulting in a final plate well DMSO concentration of 1%. The virus stock was generally diluted in infection medium (DMEM/Ham's F-12 (1:1) containing 5 µg/mL trypsin, 1% DMSO, and 1% antibiotics) to a theoretical multiplicity of infection (MOI) of 0.05. After removal of the culture medium and one washing step with PBS, the virus and compound solution were added together to the cells. In the wells used for cytotoxicity determination (uninfected cells), the virus suspension was replaced by infection medium. Each treatment was conducted in two replicates. After incubation at 37 °C, 5% CO_2 for 48 h, each well was observed in the microscope for apparent cytotoxicity, precipitate formation, or other notable abnormalities. Then, cell viability was determined by using CellTiter-Glo luminescent cell viability assay (Promega). The supernatant was removed carefully and 65 µL of the reconstituted reagent was added to each well and incubated for 15 min at room temperature under gentle shaking. Then, 60 µL of the solution was transferred to an opaque plate and luminescence (RLU) was measured by using a Synergy HT plate reader (Biotek). The

compounds were titrated on virus-infected MDCK cells and the response (RLU) was used to determine the IC_{50} value by using a 4-parameter logistic equation (GraphPad Prism) whereby the top and bottom of the curve were defined by the response (RLU) of untreated uninfected cells and untreated infected cells, respectively. The cytotoxicity of the compounds was determined by titration on uninfected MDCK cells, and the response (RLU) was used to determine the CC_{50} value by using a 4-parameter logistic equation whereby the top of the curve was defined by the response (RLU) of untreated uninfected cells.

References

1. Stanetty, C.; Czollner, L.; Koller, I.; Shah, P.; Gaware, R.; Da Cunha, T.; Odermatt, A.; Jordis, U.; Kosma, P.; Claßen-Houben, D. *Bioorg. Med. Chem.* **2010**, *18*, 7522-7541.
2. Amer, H.; Mereiter, K.; Stanetty, C.; Hofinger, A.; Czollner, L.; Beseda, I.; Jordis, U.; Kueenburg, B.; Claßen-Houben, D.; Kosma, P. *Tetrahedron* **2010**, *66*, 4390-4402.
3. Claßen-Houben, D.; Kueenburg, B.; Kosma, P.; Jordis, U.; Stanetty, C.; Szolar, O.; Wolkerstorfer, A. EP 2 258 713 A1, Dec 8, 2010.
4. MacDougall, J. M.; Zhang, X-D.; Polgar, W. E.; Khroyan, T. V.; Toll, L.; Cashman, J. R. *J. Med. Chem*. **2004**, *47*, 5809-5815.
5. Wolkerstorfer, A.; Kurz, H.; Bachhofner, N., Szolar, O. *Antiviral Res.* **2009,** *83*, 171-178.
